# The personal calculus of moral reasoning and identity in global health professions work

**Published:** 2017-04-20

**Authors:** Saleem Razack

**Affiliations:** 1 Centre for Medical Education, Faculty of Medicine, McGill University, Québec, Canada

## Abstract

In this personal essay, the author reflects on experiences in global health professions education projects, and the moral reasoning that might be required to define explicitly what constitutes ethical participation. Three interrelated notions are explored:

1. The decision to engage or not through a discussion of the concepts of safety, understanding power dynamics, and analysis of personal and institutional motivations for the project
2. The ultimate goals to promote human flourishing and improve equity, through attention to local inequities potentially experienced by either participants or colleagues from home.
3. Attention to the personal transformative potential of participation in global health professions projects.

A framework for exploring moral reasoning in global health professions education work using these three concepts is presented as one that the author has found helpful in his own work in global health professions education.

It starts with a simple form to fill out four weeks before a planned trip to Jeddah (including maybe a personal side trip to nearby Mecca). The goal of this trip is to build core competencies education capacity in residency programs in that part of Saudi Arabia. I am a pediatric intensive care physician, and was previously a pediatrics residency program director at a university in Canada. I now focus my academic and administrative work on social accountability, diversity, and equity in health professions education. I am looking forward to seeing many of my ex-residents thriving and leading in education, clinical work, and research in their respective institutions. To participate, however, I have to fill out the visa form first, and it is asking me a tough and intrusive question.

What is your religion?

My first thought is to scratch into the margins of the form that the required multiple choice answer will unfortunately have to be an essay question for me.

Do I write “Muslim, but I’m so much more,” or maybe “It’s complicated – intersected heavily by my other identities, such as belonging to a sexual minority in a mixed religion marriage with a fellow pork-avoider, both of us happily, if not mildly subversively, enjoying the odd chomp on some delicious bacon every now and then?”

The religion question and the potential visit to a sight of pilgrimage bring to my mind a flood of ancestral memories and a realization – one always has to belong to a “tribe” and does not always get to choose. My “tribe” is hard for me to define. I am the product of global movements of people going a long way back. Sugar cane plantation-based indentureship brought my people from India to the Caribbean four generations ago; economics and the promise of a better life brought my family to Canada (specifically, Toronto); residency, fellowship, and job opportunities brought me to Montreal and gave me the gift of the spoken and written words of Gallic civilization. I have trouble answering the question “Where are you from?” yet I am at home where I am.

Belonging or non-belonging is constantly negotiated and renegotiated, through a pre-determined set of social constraints. A tick-off box on a Saudi visa form is just one of those constraints.

Within that tick-off box lies a personal calculus of moral reasoning that is at the heart of the decision-making that accompanies any engagement in globalized health professions education. The problem with social constraints such as a visa form is that they are never neutral. They are imbued with power relationships, societal inequities, politics, and ideologies. They have *baggage,* and interacting with them can make a person act in ways that are inauthentic to their deeply cherished values.

What was the baggage whose heavy burden I all of a sudden appreciated filling out the visa form? In the distance I heard aunties with melodious voices singing Sufi poetry in languages they did not understand, in which connecting to God is likened to being drunk, and the divine is found in beauty. Sufi songs are considered subversive in a Wahhabist state, and when the money for the new wing of your mosque in Canada starts coming from Wahhabist charities, all of a sudden nobody sings these songs anymore. Moving to the more mundane, I also thought of female Saudi neurosurgeons we had trained at my medical school in Canada, who are not permitted to drive to their emergencies but who paradoxically are permitted to become directors of academic departments of neurosurgery, whilst performing stereotactic surgery.

With such moral complexity, how does one negotiate the doing of “good” in global health professions education?

I posit that moral reasoning in global health professions education boils down to a few questions:

1. Do I engage or not in the world, and if so, how?
2. Will my participation ultimately contribute to increasing or decreasing the flourishing and participatory potential of all people, in a just way, and with due attention to the small role I might play in the collective stewardship of human societies and our environment?
3. Am I ready to risk a change to my own perspective that exchange might bring?

## To engage or not

Positive change is a dialogic process, and the necessary conversations that produce such change can only happen through engagement. The three interrelated ideas of safety, the understanding of power dynamics, and the analysis of both personal and institutional motivations for engagement can provide a useful framework through which to develop critical consciousness when seeking to answer the question of whether to engage or not.

Safety includes concepts such as rights, rule of law, and who gets to be a fully enfranchised person, all of which can vary across the world. Risk factors for violence also vary. Violence is not always physical – for example, as an LGBT person considering travel, I must calculate physical and verbal violence risks to myself in many parts of the world, and indeed, in many parts of my own country and continent. Choosing to disclose or not can lead to a spiritual form of violence through the inherent threat to identity that the act of willfully “passing” (the pragmatic but ultimately fear-based conscious act of hiding one’s true nature from others) can bring. With respect to non-disclosure of my LGBT status, I have reasoned this by viewing duplicity and passing as nothing new in my story. It can and did easily happen in Toronto, the city I grew up in, just as it could potentially happen at points more distant.

Appreciating power flow through institutions and societies requires skills in the discernment of systemic factors. To engage others is to participate in discourse. Discourse has been defined as the conventions and understandings that regulate social institutional practices.^1^ Institutions always include people who are either empowered or disempowered within the social structures that regulate and create them. We ourselves are representatives of our own institutions when we undertake to participate in global health professions initiatives. Key questions to consider are: 1) the intrinsic value of the institution with which one is proposing to engage as a source within the host country of enhanced civil society; and 2) the diminution of hierarchies. To give an example from the Saudi context, when I give a core competencies workshop to a gender-mixed group of medical educators and lunch time comes around, will my women colleagues miss out on the social networking between myself and our male colleagues, due to the practice of gender-segregated eating? Can this be skillfully mitigated? Is my choice to eat with the men propping up unjust power structures?

In analyzing institutional motivations for engagement in global health professions education projects, I always ask myself how much of my own participation and engagement in the proposed project reflects an act of power and exploitation on the part of my own institution. This becomes much more prominent in low resource areas. For example, I have been part of projects in the former Soviet Union where different Western institutions were essentially fighting proxy academic wars for influence and prestige over unclaimed “territory” through the supposedly neutralinstrument of developing academic partnerships. In this context, it is useful to consider the degree to which the proposed intervention is exploitative versus modeling of mutual scholarly exchange.

## To be an agent of human flourishing

Academic medicine is likely composed of relatively empowered people within the societies in which it takes place. In participating in a global health professions project we must examine how the empowered group of academic health professionals with whom we are engaging have the capacity to act as a “good” within their society. The structures through which health professions education is organized and made accessible to the local population must be critically examined through the asking of a simple question: will the participation in the project contribute to greater or lesser human flourishing?

Like our own societies, the places in the world in which we choose to engage have inequities and groups that experience marginalization in health care, education, and life. Whether that be Turkic Bashkirians in majority ethnic Russian southern Siberia, or the uninsured in the United States, the personal calculus of whether to participate or not must include how one’s own participation in the health professions education intervention might nudge institutions towards greater equity. A common error of hubris is to assume that one’s foreign colleagues are not as deeply concerned about addressing inequities as you are. A particularly poignant personal example occurred in teaching advocacy to a group of dentists. Passion, indignation, and frustration were ignited in the discussion when it became known that there was no water fluoridation in a region of that country, and many in the group had been part of significant efforts to get this changed.

Another element important to my personal calculus of moral reasoning in global health professions education project participation is to consider my own colleagues back home or with me in the project. For instance, would any of them suffer significant restrictions based on their statuses as members of particular groups? In this regard, I have found that explicit discussions are always better than skirting around the issue. Such discussions can be used as a springboard to define what it might mean to be in solidarity with all marginalized people. There are no easy answers here in terms of hard definitions of whether to engage or not in situations where colleagues might have different levels of social privilege based upon their statuses as belonging to specific groups. A good test is to return to the principles of safety and agency in the promotion of human flourishing. I am reminded here of an example of my white nephew’s work as an English teacher in the Far East after he completed his university studies. He reported significant racial discrimination experienced by North American students of colour in securing jobs in Asia.

## The courage to risk being changed

To engage with another is to display the vulnerability of being potentially changed through the interaction. From my perspective, this is a huge reward and fringe benefit to participation in globalized health professions education projects. When I return to Canada after one or another health professions education project from points distant, I am always energized, and my own teaching and perspective is broadened and deepened. To appreciate another society with both different and similar problems to one’s own is to have one’s home society refracted through an analytical prism, which then can break down specific issues into a rainbow of discernible contributing factors. Through this lens of otherness, I have found that I am better able to appreciate the inequities of my own society in Canada, the constant social negotiations required to develop an integral personal identity, and indeed the existence of potential hypocrisies within my profession and academia at home.

In short, I appreciate these moments as the beautiful cacophony that is the post-modern world.

To be post-modern, as Jean-Francois Lyotard famously defined, is to be “incredulous to the grand metanarratives” that seem to define the modern world.^2^ Coming from a colonial and racialized background as I do, it has always been organically easy for me to flip the metanarratives of “Western progress” and “civilizing colonialization” on their sides. My people have not always lived these two grand metanarratives inclusively or well. The opportunity to observe the same mundane things (for example, teaching rounds on a ward) going on in different societies has allowed me to become critically conscious to local narratives of oppression and discord that must and do concern me as a physician-educator. How would a colleague from another part of the world analyze aboriginality as a determinant of health, for instance, and how would our teaching efforts to address and improve inequities experienced by Aboriginal patients or learners be seen? How does the disruption of embedding oneself, even if only briefly, in health professions education in another society, help with one’s own construction of an integral identity?

I have found the refractive potential of engaged participation in globalized health professions education particularly useful in the confrontation of the hypocrisies of my profession. The act of ticking off a religion box on a visa application shows as much about the social importance of religion in the host society as it does about my religious identity. In other countries the tick off box might be language (as in my home province of Quebec), or race (as in the US). The point is that the tick off box with its categories exists because a particular history has created the necessary conditions for it to *have* to exist. Seeing male privilege operating in overtly patriarchal societies abroad has sensitized me to my own male privilege within the more genteel patriarchy of Canadian society and my profession. Medical classes in my day had approximately 35% women students in them. These female colleagues, like me, have now become senior members of the academic medicine enterprise. Yet, if one were to attend a meeting of clinical department chairs in my university, there would not be a single female face among them.

Appreciating the impact of history on social meaning has helped me to not dichotomize global health professions education as going on “over there,” but also at home in Canada. It takes place in remote Aboriginal communities, downtown health centres, and diverse classrooms as much as it takes place “over there.” My participation in global health professions projects has allowed me to bring some of this critical dialogue to my teaching with students and residents. We can provide spaces where seemingly “just” societies can also be looked at for their inequities and also subject to a personal calculus of moral reasoning when considering engagement.

I ended up having a wonderful time in Jeddah and Mecca. It was a great pleasure to see many ex-trainees doing well. Indeed, two of my trainees, a husband and wife, graciously helped me understand the elements of the lesser version of the Muslim pilgrimage known as Umrah. I grew a little through that experience. My ex-trainees, now hosts, explained the Hajj narrative to me: at its heart it is a re-enactment of a story about a strong woman searching frantically for water, in the desert, for her son, after being cast away by the son’s Father. Through learning and reflecting on this story, I was able to appreciate the deep and proud matriarchal roots of the religion of my birth.^3^

I make no claims to any superiority of moral reasoning as I muddled through my participation in the health professions education project but I do feel that my critical consciousness and reflexivity were more finely honed and tested by the experience. Engaging in global health professions education work will likely continue to challenge my own sense of ethical action in the world, as might drivers’ licenses for female neurosurgeons or health care access for minority Bashkirians do so for others. It is in working through these ethical challenges that the seeds of possibility for transformative understandings and the potential for greater social justice might just unexpectedly lie.
